# Intranasal Dexmedetomidine for Paediatric Procedural Sedation in the Emergency Department: A Single‐Centre Retrospective Observational Cohort Study

**DOI:** 10.1111/1742-6723.70346

**Published:** 2026-09-16

**Authors:** Emma Whyte, Theophilus I. Emeto, Frances Snowden, Vinay Gangathimmaiah

**Affiliations:** ^1^ Department of Emergency Medicine Townsville Hospital Townsville Queensland Australia; ^2^ College of Medicine and Dentistry James Cook University Townsville Queensland Australia

## Abstract

**Objectives:**

To describe the use, effectiveness and safety of intranasal dexmedetomidine (IN DEX) for ED paediatric procedural sedation.

**Method:**

A retrospective cohort study was conducted over 7 months following implementation of an IN DEX guideline at Townsville University Hospital ED. Children aged 1–16 years were eligible if they received IN DEX. Outcomes of interest were sedation depth, ED length of stay (EDLOS), adverse events, procedural success and clinician/carer satisfaction.

**Results:**

A total of 114 patients were recruited. Median age was 3 years (IQR 2–5), 55.3% were male and 31.6% were Aboriginal and/or Torres Strait Islander children. Median IN DEX dose was 3.11 mcg/kg (IQR 2.81–3.93). Common indications included peripheral intravenous cannulation (35.1%), laceration repair (21.1%), burns care (13.2%), foreign body removal (11.4%) and medical imaging (8.8%). Median EDLOS was 395 min (IQR 255–579). Sedation depth was moderate (mean University Michigan Sedation Scale score 2.1 (SD 0.9), a 0–4 scale on which higher scores indicate deeper sedation; documented for 90 of 114 sedations). IN DEX was successful as a sole sedative agent in 78.1% (95% CI 69.6%–84.7%) patients, ranging from 90.0% (9/10) for medical imaging to 53.8% (7/13) for foreign body removal. Adverse events occurred in 13.2% of patients (95% CI 8.1%–20.6%), predominantly mild bradycardia. Younger age was associated with higher success rates (aOR 0.86 per year, 95% CI 0.74–0.998). No dose–response relationship was identified. Clinicians (mean 4.3/5) and parents (mean 4.5/5) reported high satisfaction, although satisfaction was documented for fewer than half of sedations and significantly more often when sedation succeeded.

**Conclusion:**

In our single‐centre study IN DEX is a well‐tolerated, moderately effective and safe agent for ED paediatric procedural sedation with higher success rates in younger patients. Large, prospective, multisite studies are necessary to establish IN DEX's safety, effectiveness and predictors of success.

## 1 Introduction

Procedural sedation is widely used in paediatric emergency departments (EDs) to facilitate distressing procedures such as imaging, wound repair and vascular access, to minimise psychological impact from a negative experience [1]. An ideal sedative agent should be effective, well tolerated, non‐invasive and safe [2]. Intranasal dexmedetomidine (IN DEX), a highly selective α2‐adrenergic receptor agonist, has emerged as a promising sedative agent in children [3, 4]. IN DEX provides anxiolysis, sedation and mild analgesia, with minimal side effects and without the need for intravenous (IV) access—a key advantage in children for whom cannulation itself is distressing and time‐consuming [5]. A significant advantage of IN DEX is the minimal respiratory depression and generally acceptable cardiovascular effects [6]. Although bradycardia and hypotension can occur, they usually do not require intervention [3, 7, 8, 9]. Needle‐free delivery of IN DEX makes it particularly suited to ED use, where time pressures, variable staff skill mix and patient distress can limit the feasibility of IV sedation.

Evidence from a growing number of studies supports the use of IN DEX in paediatric procedural sedation outside the operating room, though most studies have occurred in non‐ED settings [3, 4, 7, 9]. Karlsson et al. evaluated IN DEX delivered by radiology personnel in 1091 children undergoing MRI, reporting a 93% success rate with no critical adverse events—demonstrating its utility in diagnostic imaging [10]. A systematic review and meta‐analysis by Tervonen et al., which included 7 trials with over 730 children, found that IN DEX was effective and safe for procedural sedation for minor procedures outside of the operating theatre (diverse settings, 1 ED trial with 38 children), with a low incidence of adverse events [9]. While Baumgartner et al. identified 35 studies that included 903 paediatric patients who received dexmedetomidine (included all routes of administration, 28% IN DEX) in EDs, they found modest evidence to support efficacy and safety and highlighted the need for further research [7].

A meta‐analysis of randomised controlled trials comparing IN DEX to oral chloral hydrate for procedural sedation for medical imaging in 1846 children concluded that IN DEX had a faster onset, higher success rate with a single dose and quicker recovery [11]. Neville et al. found IN DEX performed similarly to IN Midazolam for paediatric laceration repair and was often better tolerated as it was less noxious [12]. Finnish randomised trials show IN DEX is as effective as intranasal esketamine and non‐inferior to nitrous oxide 50% for paediatric emergency procedural sedation [13, 14].

Notably, age‐related variability in the effectiveness of IN DEX has also been observed. Zhou et al. assessed IN DEX with oral midazolam in 3149 paediatric outpatients in a retrospective cohort study, reporting lower sedation success in school‐aged children compared to infants and preschoolers, highlighting the need to understand dose–response across age groups [15].

Despite the growing body of literature, most existing studies are conducted in non‐ED settings, feature single‐centre designs, have small sample sizes and employ heterogeneous methodologies. Furthermore, many fail to report on ED‐specific outcomes, such as sedation onset time, duration or length of stay. There are no published Australian studies about the safety and effectiveness of IN DEX for ED paediatric procedural sedation. Uncertainties persist around variable onset time, depth of sedation and haemodynamic effects such as bradycardia or hypotension. The aim of this study was to address these uncertainties by describing use, effectiveness and safety of IN DEX for paediatric procedural sedation at an Australian ED. The study also sought to explore predictors of procedural success, adverse events and emergency department length of stay (EDLOS).

## 2 Methods

This was a retrospective, observational cohort study conducted at Townsville University Hospital ED, a major referral mixed Queensland ED with an annual census of 97,259 (including 20% paediatric) in FY 2024–2025. An IN DEX paediatric procedural sedation guideline (Supporting Information: Appendix 1) was developed and implemented in September 2024. The guideline implementation was paired with ED clinician (medical and nursing) education and a standardised IN DEX procedural sedation note (Supporting Information: Appendix 2).

Children aged 1–16 years were eligible for IN DEX (2–4 mcg/kg) procedural sedation for the following indications: Painless procedures requiring sedation (e.g., CT/MRI scans), vascular access and wound repair procedures provided additional analgesics are used (e.g., topical anaesthetics). Contraindications in the guideline were age under 12 months, known hypersensitivity to dexmedetomidine/clonidine and known cardiac/neurological comorbidities. Patients were selected for IN DEX sedation at the discretion of the treating emergency physicians. Details of sedation were entered prospectively into the standardised note by treating medical and nursing clinicians. Outcomes of interest were sedation depth, EDLOS, adverse events (safety outcome), procedural success (effectiveness outcome) and clinician/carer satisfaction. Sedation depth was measured using the University of Michigan Sedation Scale (UMSS), scored from 0 to 4: 0, awake and alert; 1, minimally sedated; 2, moderately sedated; 3, deeply sedated; 4, unarousable. Higher scores indicate deeper sedation, and the deepest score reached during each sedation episode was recorded. Procedural success was defined as successful completion of procedure with IN DEX as the sole sedative agent. Adverse events were defined as any event requiring intervention or a vital sign outside of 95th centiles for patients age. Clinician/carer satisfaction was measured using a Likert scale of 1–5 (1‐very unsatisfied, 2‐unsatisfied, 3‐neutral, 4‐satisfied, 5‐very satisfied).

A list of all children aged 0–16 years who received IN DEX from 1 September 2024 to 30 March 2025 was sourced from Townsville University Hospital ED business practice improvement officer. The duration was chosen to enable data analysis for at least 100 patients. The electronic medical record (iEMR, Cerner) was then interrogated by the lead author to ascertain eligibility and collect data into a standardised data collection form (Microsoft Excel). Data were collected on demographics, dose, indication, sedation depth, physical observations, adverse events, procedural success and clinician/carer satisfaction.

Standard chart review guidelines were followed to minimise bias [16]. Predefined eligibility criteria and clearly specified variables were established a priori, supported by a standardised data abstraction tool and coding guide. All eligible patients who received dexmedetomidine were included. Data abstraction was performed by the lead author. The coding guide is provided as Supporting Information: Appendix 3.

All analyses were performed using Stata 19.0 (StataCorp, College Station, TX). A sample size calculation was not performed for this descriptive study. Continuous variables were described using means with standard deviations for normally distributed data and medians with interquartile ranges for skewed distributions. Normality was assessed using graphical methods and Shapiro–Wilk tests. Categorical variables were reported as frequencies and percentages. Wilson score confidence intervals were calculated for all proportions, providing superior coverage compared to Wald intervals, particularly for proportions near 0 or 1 and smaller sample sizes [17, 18, 19]. Chi‐square tests were used for categorical comparisons, with Fisher's exact tests employed when expected cell counts were less than 5. The Mann–Whitney *U* test was used for comparing skewed continuous outcomes between groups. Logistic regression was used for binary outcomes, with odds ratios and 95% confidence intervals reported. Linear regression was performed on log‐transformed EDLOS given non‐normality; results are presented as regression coefficients which can be exponentiated to obtain multiplicative effects. Model fit was assessed using Hosmer–Lemeshow goodness‐of‐fit tests and area under the receiver operating characteristic curve for logistic models, and *R* ^2^ with variance inflation factors for linear models [20]. Statistical significance was defined as two‐sided *p* < 0.05. No adjustments for multiple comparisons were made given the exploratory and hypothesis‐generating nature of subgroup analyses. Predictors of each key outcome; sedation success, any adverse event and EDLOS, were examined using a common set of candidate variables (age, weight, dose, sex, First Nations status, concurrent fentanyl, any medical history, after‐hours presentation, weekend presentation and procedure group), reported univariably for all three outcomes in Table S1. Multivariable models were pre‐specified and were not revised after inspection of the data: sedation success was modelled adjusting for age, dose, sex and procedure group; adverse events adjusting for age, dose and sex; and ln(ED length of stay) adjusting for age, dose, sedation success, adverse event, procedure group and after‐hours presentation. Linear regression coefficients were exponentiated and are reported as the percentage change in the geometric mean EDLOS. Multivariable models were deliberately restricted to the pre‐specified covariates because the number of events per variable was 5.0 for both logistic models (25 sedation failures for 5 degrees of freedom; 15 adverse events for 3 degrees of freedom), below the conventional minimum of 10; univariable associations are therefore reported as exploratory. Because the adverse‐event model was sparse, Firth penalised‐likelihood logistic regression was fitted as a sensitivity analysis (Table S2). No adjustment for multiplicity was applied to the primary reporting, but Holm‐adjusted *p* values were computed for the univariable adverse‐event screen to identify chance findings.

The mechanism of missingness was examined rather than assumed. For each incompletely documented outcome, documentation completeness was compared between successful and unsuccessful sedations using Fisher exact tests, and the odds of non‐documentation were modelled adjusting for age, dose, sex, after‐hours presentation and adverse event (Table S3, Figure S2). Where missingness was associated with an observed outcome, satisfaction estimates were re‐computed under a missing‐at‐random assumption conditional on sedation success (success‐standardised to the full cohort) and bounded by extreme‐case analyses in which all undocumented responses were assumed satisfied and then all assumed dissatisfied (Table S4).

This study was approved as non‐research by Townsville Hospital and Health Service Audit Quality and Innovation Review panel (THHSAQUIRE1825). The study report adheres to Strengthening the Reporting of Observational Studies in Epidemiology (STROBE) guidelines (Supporting Information: Appendix 4).

## 3 Results

All 114 patients who met eligibility criteria were recruited. Patient characteristics are presented in Table 1.

**TABLE 1:** Baseline characteristics, dose and indications for children (*n* = 114) aged 1–16 years who had procedural sedation with intranasal dexmedetomidine (IN DEX) at Townsville University Hospital Emergency Department.

Characteristics	Value
Demographics	
Age, years—median (IQR)	3 (2–5)
Age group—*n* (%)	
1–2 years	44 (38.6)
3–5 years	42 (36.8)
6–11 years	26 (22.8)
12–16 years	2 (1.8)
Male sex—*n* (%)	63 (55.3)
Weight, kg—median (IQR)	15.9 (12.8–22.2)
Indigenous Australian—*n* (%)	36 (31.6)
Dose	
Dose, mcg/kg—median (IQR)	3.11 (2.81–3.93)
Dose category—*n* (%)	
< 3 mcg/kg	49 (43.0)
3–4 mcg/kg	54 (47.4)
> 4 mcg/kg	11 (9.6)
Indications—*n* (%)	
IV Cannulation	40 (35.1)
Laceration repair	24 (21.1)
Burns debridement and dressing	15 (13.2)
Foreign body removal	13 (11.4)
Medical imaging	10 (8.8)
Wound care	3 (2.6)
Other/examination^a^	9 (7.9)

^a^ Examination (3), eye examination (3), backslab application (1), enema (1) and ultrasound (1).

**FIGURE 1:**
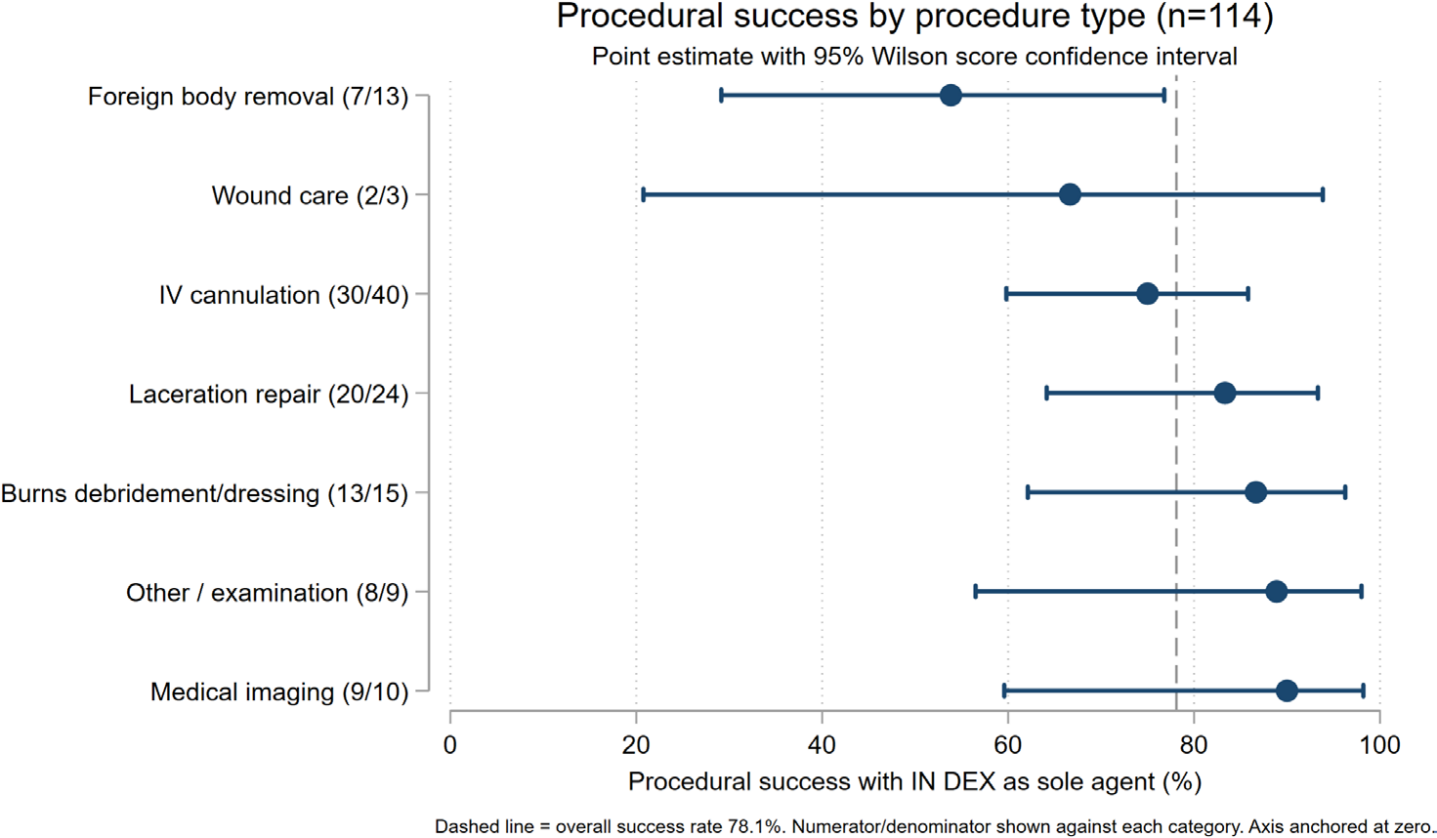
Procedural success by procedure type, with 95% Wilson score confidence intervals (*n* = 114). Point estimates ordered by observed success rate. The dashed vertical line marks the overall success rate of 78.1%. Numerators and denominators are shown against each category. The percentage axis is anchored at zero. Wide intervals for wound care (*n* = 3), other/examination (*n* = 9), medical imaging (*n* = 10) and foreign body removal (*n* = 13) reflect small category sizes.

Procedural success was achieved in 89 of 114 patients (78.1%; 95% CI 69.6%–84.7%). Success by procedure type is presented in Table 2 and Figure 1. Success was highest for medical imaging (9/10; 90.0%, 95% CI 59.6%–98.2%) and lowest for foreign body removal (7/13; 53.8%, 95% CI 29.1%–76.8%), with intermediate rates for burns debridement and dressing (13/15; 86.7%), laceration repair (20/24; 83.3%) and IV cannulation (30/40; 75.0%). The overall test across the seven procedure categories was not statistically significant (Fisher exact *p* = 0.328), and in the pre‐specified multivariable model procedure group was not associated with success after adjustment for age, dose and sex (Table 3; Table S1, Panel A). In an unplanned contrast prompted by peer review, foreign body removal compared with all other procedures combined gave an odds ratio of 0.27 (95% CI 0.08–0.90; Fisher exact *p* = 0.036); this is one of seven possible procedure‐versus‐rest comparisons and does not meet a Bonferroni‐corrected threshold of *α* = 0.007, and the confidence interval around the foreign‐body‐removal success rate is wide. These procedure‐specific estimates are therefore descriptive and hypothesis‐generating. Subgroup analyses revealed no significant differences in success rates across dose categories (< 3 mcg/kg: 75.5%; 3–4 mcg/kg: 79.6%; > 4 mcg/kg: 81.8%; *p* = 0.838) or gender (males 77.8% vs. females 78.4%; *p* = 0.933). Lower success rates were observed in older children (1–2 years: 84.1%; 3–5 years: 83.3%; 6–11 years: 61.5%; 12–16 years: 50.0%; *p* = 0.083), though this did not reach statistical significance.

**TABLE 2:** Procedural success, adverse events and ED length of stay by procedure type (*n* = 114).

Procedure type	*n*	Procedural success, *n* (%)	95% CI	Adverse event, *n* (%)	EDLOS, median (IQR), min
Medical imaging	10	9/10 (90.0)	59.6–98.2	2/10 (20.0)	434 (397–688)
IV cannulation	40	30/40 (75.0)	59.8–85.8	7/40 (17.5)	474 (304–1094)
Laceration repair	24	20/24 (83.3)	64.1–93.3	2/24 (8.3)	310 (240–456)
Burns debridement/dressing	15	13/15 (86.7)	62.1–96.3	3/15 (20.0)	402 (240–1703)
Wound care	3	2/3 (66.7)	20.8–93.9	0/3 (0.0)	240 (121–1813)
Foreign body removal	13	7/13 (53.8)	29.1–76.8	1/13 (7.7)	275 (219–464)
Other/examination	9	8/9 (88.9)	56.5–98.0	0/9 (0.0)	364 (255–474)
All procedures	114	89/114 (78.1)	69.6–84.7	15/114 (13.2)	395 (255–579)
*p* across procedure types		0.328		0.726	0.080

*Note:* Procedural success is defined as completion of the procedure with IN DEX as the sole sedative agent. Wilson score 95% confidence intervals are reported for all proportions. *p* values are from the Fisher exact test (procedural success, adverse events) and the Kruskal–Wallis test (EDLOS) across all seven procedure categories. Several categories contain fewer than 15 patients and the corresponding confidence intervals are wide; between‐procedure comparisons are descriptive and hypothesis‐generating only.

Abbreviations: CI, confidence interval; ED, emergency department; EDLOS, emergency department length of stay; IN DEX, intranasal dexmedetomidine; IQR, interquartile range.

**TABLE 3:** Predictors of sedation success—Logistic regression analysis.

Predictor	OR (95% CI)	*p*	aOR (95% CI)^a^
Age (per year)	0.86 (0.74–0.99)	0.037	0.86 (0.74–0.998)^†^
Dose (per mcg/kg)	0.85 (0.45–1.59)	0.611	0.94 (0.48–1.81)
Male sex	0.96 (0.39–2.35)	0.933	0.88 (0.34–2.25)
Weight (per kg)	0.95 (0.91–0.99)	0.020	—
After‐hours presentation	1.14 (0.44–2.93)	0.792	—

Abbreviations: aOR, adjusted odds ratio; CI, confidence interval; OR, odds ratio.

^a^ Adjusted for age, dose, sex and indication.

^†^ *p* = 0.048; 95% CI 0.74–0.998 (borderline significant).

The adverse event rate was 13.2% (15/114; 95% CI 8.1%–20.6%). Bradycardia was the most common adverse event, occurring in 15 patients (13.2%; 95% CI 8.1%–20.6%), followed by hypotension (*n* = 2 (1.8%)) and transient oxygen desaturation (*n* = 1 (0.9%)). Two patients (1.8%) received interventions (tactile stimulation only). No pharmacological interventions, airway manoeuvres, or resuscitation measures were required. All adverse events were self‐limiting and resolved without sequelae.

Adverse event rates did not differ significantly by dose category (< 3 mcg/kg: 12.2%; 3–4 mcg/kg: 14.8%; > 4 mcg/kg: 9.1%; *p* = 0.850). A non‐significant lower adverse event rate was observed in the youngest age group (1–2 years: 4.5% vs. 3–5 years: 21.4% vs. 6–11 years: 15.4%; *p* = 0.098).

A UMSS score was documented for 90 of 114 sedations (78.9%). The mean deepest score was 2.1 (SD 0.9), indicating moderate sedation overall. The distribution was: 4 sedations (4.4%) remained awake and alert (UMSS 0), 15 (16.7%) were minimally sedated (UMSS 1), 38 (42.2%) moderately sedated (UMSS 2), 32 (35.6%) deeply sedated (UMSS 3) and 1 (1.1%) unarousable (UMSS 4). The full distribution is shown in Figure S3. Sedation was deeper in successful than unsuccessful sedations (mean 2.32, SD 0.76 vs. 1.29, SD 0.77; *p* < 0.001; Table S4).

Median EDLOS was 395 min (IQR 255–579). EDLOS did not differ significantly with procedural success (successful vs. unsuccessful: 390 vs. 469 min; *p* = 0.316) or adverse events (presence vs. absence: 461 vs. 381 min).

Clinician satisfaction was documented for 45 of 114 sedations (39.5%) and parent/carer satisfaction for 41 of 114 (36.0%). Among those documented, mean satisfaction scores were 4.3 (SD 1.0; 95% CI 4.0–4.6) for clinicians and 4.5 (SD 0.8; 95% CI 4.2–4.7) for parents, and a score of 4 or higher was reported by 80.0% (95% CI 66.2%–89.1%) of clinicians and 87.8% (95% CI 74.5%–94.7%) of parents (Table 4). Because documentation of satisfaction was itself associated with sedation success (see Missing Data), these available‐case estimates are likely to be optimistic; under a missing‐at‐random assumption conditional on sedation success the corresponding estimates were 4.1% and 75.7% for clinicians and 4.3% and 82.7% for parents (Table S4). Univariate logistic regression identified age and weight as significant predictors of sedation success (Table 3). In multivariable analysis adjusting for age, dose, sex and indication, age remained the only statistically significant predictor (adjusted OR 0.86; 95% CI 0.74–0.998; *p* = 0.048). The model showed modest discrimination (area under the receiver operating characteristic curve 0.65) with adequate calibration (Hosmer–Lemeshow *p* = 0.465). Predictors of all three key outcomes are presented together in Table S1 and Figure S1.

**TABLE 4:** Outcomes for children (*n* = 114) aged 1–16 years who had procedural sedation with intranasal dexmedetomidine (IN DEX) at Townsville University Hospital Emergency Department.

Outcome	*n*/*N* (%)	95% CI
Sedation success (sole agent)	89/114 (78.1)	69.6–84.7
Any adverse event	15/114 (13.2)	8.1–20.6
Specific adverse events		
Bradycardia	15/114 (13.2)	8.1–20.6
Hypotension	2/114 (1.8)	0.4–6.2
Desaturation	1/114 (0.9)	0.1–4.8
Intervention required	2/114 (1.8)	0.4–6.2
Procedure not completed with IN DEX	25/114 (21.9)	15.3–30.3
Nitrous oxide rescue	9/114 (7.9)	
Ketamine rescue	8/114 (7.0)	
Procedure in operating theatre	4/114 (3.5)	
Procedure not completed	2/114 (1.8)	
Procedure completed without sedation	2/114 (1.8)	

Outcome	*n* (%)	Mean (SD)	Median (IQR)
UMSS (deepest)	90 (78.9)	2.1 (0.9)	
ED LOS (minutes)	114 (100)		395 (255–579)
Clinician satisfaction (1–5)	45 (39.4)	4.3 (1.0)	
Parent satisfaction (1–5)	41 (35.9)	4.5 (0.8)	

*Note:* Wilson score confidence intervals used for proportions. Adverse‐event categories are not mutually exclusive: all 15 patients with an adverse event had bradycardia, the 2 patients with hypotension and the 1 patient with desaturation also had bradycardia and 3 patients had more than one adverse‐event type. Denominators for UMSS and satisfaction reflect available‐case analysis; see Tables S3 and S4.

Abbreviations: CI, confidence interval; ED LOS, emergency department length of stay; IQR, interquartile range; SD, standard deviation; UMSS, University of Michigan Sedation Scale.

No candidate predictor was associated with adverse events once multiple testing was taken into account, and the pre‐specified multivariable model including age, dose and sex showed no significant predictors (likelihood ratio test *p* = 0.764); full results are presented in Table S1, Panel B. Weekend presentation was nominally associated with adverse events (7/25 vs. 8/89; OR 3.94, 95% CI 1.26–12.26; *p* = 0.018), but this was 1 of 11 univariable tests, did not survive Holm correction (adjusted *p* = 0.198), was not explained by age, dose, sex or procedure type, and has no plausible pharmacological basis; it is most consistent with a chance finding. Estimates were materially unchanged under Firth penalised likelihood (Table S2). Given the low event rate (*n* = 15) and 5.0 events per variable, power to detect predictors was limited.

In the pre‐specified multivariable model (Table S1, Panel C) after‐hours presentation was associated with a longer stay (86.2% longer geometric mean EDLOS, 95% CI 40.7–146.4%; *p* < 0.001). Procedure group also influenced EDLOS, with procedures (laceration repair, wound care, burns care) associated with a shorter stay than medical imaging (39.8% shorter, 95% CI 63.2% shorter to 1.4% shorter; *p* = 0.044). Sedation success, adverse events, age and dose were not associated with EDLOS in the adjusted model. The model explained a modest proportion of the variance (*R* ^2^ 0.215, adjusted *R* ^2^ 0.163), with no evidence of collinearity (maximum variance inflation factor 3.52). Sensitivity analyses demonstrated higher success in younger children but no difference across doses or indications (Table 5).

**TABLE 5:** Sensitivity analyses—Sedation success by subgroup.

Analysis population	*N*	Success (%)	95% CI
Age < 3 years	44	84.1	70.6–92.1
Age ≥ 3 years	70	74.3	63.0–83.1
Dose ≥ 3.5 mcg/kg	46	76.1	62.1–86.1
Dose < 3.5 mcg/kg	68	79.4	68.4–87.3
Excluding imaging procedures	104	76.9	67.9–84.0

Abbreviation: CI, confidence interval.

All variables entering the analyses of the three key outcomes: procedural success, adverse events, EDLOS, age, weight, dose, sex, procedure type and time of presentation were complete for all 114 patients. Missingness was confined to three secondary outcomes: depth of sedation (24/114, 21.1%), clinician satisfaction (69/114, 60.5%) and parent/carer satisfaction (73/114, 64.0%) (Table S3). The mechanism differed between these outcomes. Documentation of depth of sedation did not differ significantly by sedation success (82.0% vs. 68.0%, *p* = 0.165), consistent with missingness driven by documentation workload rather than by the outcome. In contrast, satisfaction was recorded substantially more often when sedation had been successful (clinician 46.1% vs. 16.0%, *p* = 0.010; parent/carer 41.6% vs. 16.0%, *p* = 0.020), and these associations persisted after adjustment for age, dose, sex, after‐hours presentation and adverse event (Figure S2). The satisfaction data are therefore not missing completely at random, and the reported satisfaction estimates should be interpreted as describing a favourably selected subgroup. Sensitivity analyses under a missing‐at‐random assumption conditional on sedation success attenuated but did not reverse the estimates, whereas extreme‐case bounds were uninformative (clinician 31.6%–92.1%; parent/carer 31.6%–95.6%) (Table S4).

## 4 Discussion

In this retrospective cohort study, IN DEX demonstrated moderate procedural success with a favourable safety profile as a sole sedative agent for paediatric ED procedures. Procedural success was more likely in younger children, and overall clinician and parent satisfaction was high despite variable sedation depth.

The findings of this study should be interpreted as exploratory and hypothesis generating. The observed success rate of 78.1% is consistent with prior literature, which reports high success rates for nonpainful procedures such as imaging, but lower success rates for painful interventions [3, 4, 7, 8, 9]. Our procedure‐specific data are consistent with that gradient (Table S5): success was 90.0% for medical imaging but only 53.8% for foreign body removal, the most physically stimulating procedure in the cohort and the only category in which fewer than two‐thirds of procedures were completed with IN DEX alone. This difference did not reach conventional significance after accounting for the seven possible procedure comparisons, and the confidence intervals are wide, so it must be regarded as a hypothesis rather than an established effect. It is also partly confounded by age, since foreign body removal was undertaken in older children and age was the only significant predictor of success in the adjusted model. Nonetheless the pattern is clinically coherent and potentially actionable: where IN DEX is being considered as a sole agent for foreign body removal, clinicians should anticipate a meaningful probability of needing rescue sedation and should plan pre‐emptively for adjunctive analgesia or an alternative strategy. The absence of standardised pain or distress measures limits our ability to test the underlying mechanism, and procedural stimulus intensity remains a plausible unmeasured confounder.

No adverse events requiring intervention beyond physical stimulation were observed. There was an episode of transient desaturation included; however, the patient went on to be admitted for community acquired pneumonia and have supplemental oxygen therapy, which was more likely the cause of desaturation. This provides real‐world evidence supporting the safety of IN DEX for ED paediatric procedural sedation. Reported adverse events were limited to deviations in vital signs beyond age‐adjusted 95th centiles, which may have limited clinical relevance from a patient‐centred perspective. This safety profile is consistent with previous studies reporting low rates of clinically significant adverse events associated with IN DEX use [3, 4, 7, 8, 9].

IN DEX appeared to have a variable duration of action, with prolonged sedative effects observed in some patients. However, substantial missing data limited reliable assessment of time to onset, duration of action and depth of sedation. Documentation of sedation depth was inconsistent. Despite this, carer satisfaction appeared high without a clear relationship to sedation depth. These findings suggest that satisfaction may be influenced by factors other than sedation depth alone, such as communication, perceived comfort, or procedural outcome.

Our study has a number of potential limitations. First, the single‐centre design precludes generalisability. Second, the small sample size limits power to detect predictors of success and adverse events, particularly given the low rate of adverse events observed. Third, the observed association between younger age and higher procedural success was of borderline statistical significance and the modest discriminative ability of the regression model suggests that additional unmeasured variables likely influence sedation success. Small numbers within age and procedure subgroups further limit the strength of conclusions regarding these associations.

Fourth, missing data introduce potential information bias. Satisfaction scores were documented for fewer than half of sedations and, importantly, were recorded substantially more often when sedation had been successful; the reported satisfaction estimates therefore describe a favourably selected subgroup and are likely to be optimistic, although sensitivity analyses suggest the magnitude of the bias is modest under a missing‐at‐random assumption. Documentation of depth of sedation, by contrast, showed no association with the outcome. This missingness likely reflects operational constraints on documentation during busy ED shifts. Fifth, the absence of a comparator group limits inference regarding the relative effectiveness of IN DEX compared with alternative sedation strategies. Finally, the retrospective design limited standardisation of data collection and outcome assessment, introducing potential for selection bias and residual confounding.

In addition, the number of events per variable was 5.0 for both the sedation‐success and adverse‐event models, below the conventional minimum of 10, so all multivariable estimates should be regarded as imprecise and the absence of significant predictors of adverse events cannot be taken as evidence of their absence. Procedure‐specific success rates are based on as few as three patients in some categories, and the comparisons between procedure types were not pre‐specified; they are reported to inform the design of prospective studies rather than to support practice change on their own.

Finally, our definition of procedural success may also have influenced age‐related findings. Younger children may be more readily physically restrained, allowing procedural completion despite suboptimal sedation depth. This may have inflated apparent success rates in younger age groups.

IN DEX is a promising agent for ED paediatric procedural sedation due to ease of administration and favourable tolerability. While its time to onset may be perceived as a limitation in emergent situations, this aligns well with the onset of topical local anaesthetics, making IN DEX a feasible option for needle‐based procedures.

Application of consensus‐based definitions for adverse events in procedural sedation research in future studies would enhance comparability across studies and improve interpretation of safety outcomes [21]. Prospective, multicentre studies are needed to better characterise the pharmacodynamic profile of IN DEX in the emergency setting, including time to onset, peak effect and duration of action, and to identify predictors of procedural success and adverse events. Further research should also explore the role of IN DEX in combination with other sedative agents and incorporate validated pain/distress measures to improve assessment of sedation effectiveness and patient‐centred outcomes.

## 5 Conclusion

IN DEX demonstrated moderate effectiveness and favourable safety for paediatric ED procedures. Variable sedation depth and modest success rate, particularly for painful procedures, highlight the need for careful patient/procedure selection and consideration of adjunctive analgesia or alternative sedation strategies. Prospective, multi‐centre studies are needed to define the role of IN DEX in paediatric emergency procedural sedation.

## Funding

The authors have nothing to report.

## Conflicts of Interest

The authors declare no conflicts of interest.

## Supporting information


**Table S1:** Univariable and multivariable predictors of the three key outcomes: sedation success, adverse events, and ED length of stay (*n* = 114).
**Table S2:** Firth penalised‐likelihood sensitivity analysis for the adverse‐event model.
**Table S3:** Extent and mechanism of missing data (*n* = 114).
**Table S4:** Sensitivity of satisfaction estimates to alternative missing‐data assumptions.
**Table S5:** Procedural success and adverse events by pre‐specified procedure group (*n* = 114).
**Figure S1:** Adjusted predictors of the three key outcomes (*n* = 114).
**Figure S2:** Completeness of outcome documentation by sedation success.
**Figure S3:** Distribution of the deepest UMSS score.

## Data Availability

The data that support the findings of this study are available on request from the corresponding author. The data are not publicly available due to privacy or ethical restrictions.
