# Supplementary material for: Intranasal Dexmedetomidine for Paediatric Procedural Sedation in the Emergency Department: A Single‐Centre Retrospective Observational Cohort Study
Source: Emerg Med Australas. 2026 Sep 16;38(5):e70346. doi: 10.1111/1742-6723.70346 (PMC13583133; doi:10.1111/1742-6723.70346)
Supplement: Supplementary file 1 [file EMM-38-0-s001.docx]

## **Supplementary Material**

## ***Intranasal Dexmedetomidine for Paediatric Procedural Sedation in the Emergency Department: A Single-Centre Retrospective Observational Cohort Study***

## ***Whyte E, Emeto TI, Snowden F, Gangathimmaiah V.***

## Appendix 1. IN DEX Emergency Department Guideline

**Emergency Department Guideline**	**MSG.ED.3.1.5**

| Title: | Paediatrics |
| --- | --- |
|  | |
| Description: | Intranasal Dexmedetomidine (IN DEX) for Paediatric Procedural Sedation |
|  | |
| Target Audience: | Emergency Department Clinical Staff |

Alerts/Special Considerations

- For use in children >12months undergoing non-painful procedures with no significant cardiac or neurological comorbidity.
- Requires continuous O2 sats and HR monitoring.
- Any change in HR of more than 20% (or a HR outside normal range for age) requires BP to be checked.
- Although rarely clinically significant, dexmedetomidine can cause bradycardia and hypotension (can also cause initial transient hypertension). Do not use in cases of haemodynamic abnormality.
- Document with INDEX auto text in iEMR This feedback will help refine the use of IN DEX and optimize care.

#### **Drug Procedure:**

<https://qheps.health.qld.gov.au/__data/assets/pdf_file/0029/3190934/dexmedetomidine-intranasal-picu-ed.pdf>

#### **Introduction**

Intranasal Dexmedetomidine (IN DEX) is a non-invasive sedative option for paediatric procedural sedation, it can be used for imaging and minor procedures in the emergency department (ED). Its properties include sedation and anxiolysis via alpha-2 adrenergic receptor agonism (decreasing sympathetic tone), making it an ideal choice due to its minimal respiratory depression and good cardiovascular profile. However, it is important to be mindful of potential bradycardia and hypotension during its use. It produces a sedated but rousable patient.

Intranasal Dexmedetomidine (IN DEX) has been used in intensive care and anaesthetic settings for some time but is **off-label** for use in paediatric patients and will therefore **require informed consent from guardian**.

#### **Indications**

- **Paediatric** patients aged 12 months to 15 years.
- **Painless procedures** requiring sedation (e.g., **CT/MRI scans**).
- **Vascular access** and **wound repair procedures** provided additional analgesics are used (e.g., topical anesthetics).

#### **Contraindications**

- Age **under 12 months**. (relative CI)
- Known **hypersensitivity** to Dexmedetomidine or clonidine.
- **Cardiac conditions** or known **neurological comorbidities.**

Precautions:

- In patients with **hepatic dysfunction**, a dose reduction may be necessary due to slower metabolism of Dexmedetomidine.

#### **Dosage and Administration**

[CHQ-NSS-51022 Intranasal Medication Administration (health.qld.gov.au)](https://www.childrens.health.qld.gov.au/__data/assets/pdf_file/0034/179683/Intranasal-Medication-Administration.pdf)

***Presentation:*** Dexmedetomidine is available as a **200 mcg/2 mL** vial.

***Dose:* 2–4 mcg/kg**, rounded up to the nearest 5 mcg (maximum dose: **200 mcg**). If starting dose less than 4mcg/kg can top up to no more than 4mcg/kg if insufficient response at 30min.

##### ***Administration:***

- Use a **Mucosal Atomizer Device (MAD)**.
- Divide the dose evenly between both nostrils.
- Ensure the dose is administered **30 minutes before the procedure** for optimal sedation.

##### ***Steps:***

- Draw up the calculated dose plus 0.1 mL to account for dead space in the atomizer.
- Attach the MAD Nasal device and administer half the dose per nostril.
- Ensure that the patient is positioned appropriately (sitting or lying flat) and encourage older children to blow their nose beforehand to optimize absorption.
- **Do not instruct children ‘to take a big sniff**’. Taking a big sniff can displace medication into the mouth rendering it less effective.
- The ideal volume is **0.2–0.3 mL** per nostril; avoid exceeding **1 mL** to prevent drug loss or delayed absorption if the solution drips down the throat.

#### **Onset, Duration, and Monitoring**

***Onset:*** 10-20 minutes.

***Peak Effect:*** 30-60 minutes.

***Duration:*** 60-120 minutes. (dose dependent)

##### ***Monitoring:***

- Record Baseline full set of vital signs. *Reconsider Dexmedetomidine if pre-existing bradycardia or hypotension is present.*
- Continuous **heart rate (HR)** and **oxygen saturation (SpO2)** monitoring is required throughout sedation and recovery.
- Record vital signs every **15 min**. BP not required except at 15min and 1hr unless there is a change of more than 20% in HR or HR outside normal range for age.
- **Blood pressure (BP)** monitoring: Perform baseline checks and recheck **15 minutes** post-administration and at **1 hour** unless clinically indicated. *Less frequent BP monitoring is suggested to avoid waking the patient unnecessarily during sedation but HR vigilance is required while BP is not being regularly checked.*
- Most patients recover to baseline within 90 minutes. A full set of vitals should be checked at recovery.

#### **Adverse Effects**

**Common:** Bradycardia, mild hypotension, nausea, dry mouth.

**Rare:** Arrhythmia, significant hypotension, headache, fever, confusion, dizziness, visual disturbances

While IN DEX has a **favorable respiratory profile**, it is critical to remain vigilant for **transient bradycardia** and **hypotension**, especially during the first hour post-administration

#### **Treatment of Complications**

- Call for senior help
- Stop procedure
- Lie flat or in Trendelenburg position
- Establish IV access and give fluid bolus in first instance for clinically significant hypotension
- If fluid resistance, consider vasopressors +/- atropine (if associated bradycardia)

#### **Documentation**

Use **INDEX auto text** in iEMR to document

- Dose and time of administration
- Time of sedation onset
- Time procedure commenced
- Sedation score
- Vital signs before, during, and after the procedure
- Any adverse events or interventions
- Time to recovery and patient/parent satisfaction
- Clinician satisfaction

## Appendix 2. – iEMR Documentation

Intranasal Dexmedetomidine Sedation Record - for use in conjunction with the THHS medication guideline

Indication: _▼

Informed Consent: _▼

Dose:

Time given:

Time effect noticed:

Time procedure commenced:

Sedation score (at commencement of procedure): _▼

Deepest level of sedation achieved: _▼

Time of deepest sedation:

Time of recovery to baseline:

Full set of vital signs pre-sedation and then at 15min, and 60mins post administration of sedation.

Additional vital signs NOT including BP at 30mins and 45mins.

*If HR abnormal at any time a BP must be checked.

Additional vital signs as clinically indicated during procedure can be documented in interactive view.

| Time point | BP | HR | SpO2 | RR | Temp |
| --- | --- | --- | --- | --- | --- |
| Pre-sedation  Baseline |  |  |  |  |  |
| 15mins |  |  |  |  |  |
| 30mins |  |  |  |  |  |
| 45mins |  |  |  |  |  |
| 60mins |  |  |  |  |  |
| post sedation |  |  |  |  |  |

Successful completion of procedure: _▼

Adverse events: _▼

Parent/Carer satisfaction: _▼

Clinician satisfaction: _▼

Comments:

## Appendix 3. – Coding guide

The coding guide specified the following rules a priori for missing and conflicting information.

**Source hierarchy for conflicting information.**

Where the same variable appeared in more than one location in the record, precedence was given to (1) the standardised IN DEX procedural sedation note, then (2) the contemporaneous nursing observation chart, then (3) the medical progress note, then (4) the discharge summary. Where a conflict persisted within the same tier, the value less favourable to IN DEX was taken; the lighter sedation score, the presence rather than the absence of an adverse event, and the longer ED length of stay.

**Procedural success.** Coded from documented completion of the procedure together with documented administration of any additional sedative agent. Where the sedation note and the procedure note disagreed, the medication administration record was treated as adjudicating.

Adverse events. Defined by pre-specified physiological thresholds (values beyond age-adjusted 95th centiles) applied to recorded observations. Absence of a documented adverse event in a patient with a complete observation set was coded as absence of the event; patients with an incomplete observation set were not imputed as event-free, and no such patient occurred.

**Physiological observations.** Where a scheduled observation was not recorded but adjacent observations were available and no intervening clinical change was documented, the last observation was carried forward. This rule was applied only to routine vital-sign time series. It was not applied to any outcome variable or to depth of sedation.

**No imputation of outcomes.** No single or multiple imputation was performed for any outcome. Outcomes with incomplete documentation (depth of sedation, clinician and carer satisfaction) were analysed on an available-case basis with the denominator reported for every estimate.

**Eligibility.** Records with insufficient documentation to establish eligibility would have been excluded and the number reported; no record required exclusion on this basis.

Appendix 4 – STROBE Checklist

STROBE Statement—checklist of items that should be included in reports of observational studies

|  | **Item No.** | **Recommendation** | **Page**  **No.** |  |
| --- | --- | --- | --- | --- |
| **Title and abstract** | 1 | (*a*) Indicate the study’s design with a commonly used term in the title or the abstract | 1 |  |
|  |  | (*b*) Provide in the abstract an informative and balanced summary of what was done and what was found | 1 |  |
| **Introduction** | | | |  |
| Background/rationale | 2 | Explain the scientific background and rationale for the investigation being reported | 2 |  |
| Objectives | 3 | State specific objectives, including any prespecified hypotheses | 3 |  |
| **Methods** | | | |  |
| Study design | 4 | Present key elements of study design early in the paper | 3 |  |
| Setting | 5 | Describe the setting, locations, and relevant dates, including periods of recruitment, exposure, follow-up, and data collection | 4 |  |
| Participants | 6 | (*a*) *Cohort study*—Give the eligibility criteria, and the sources and methods of selection of participants. Describe methods of follow-up  *Case-control study*—Give the eligibility criteria, and the sources and methods of case ascertainment and control selection. Give the rationale for the choice of cases and controls  *Cross-sectional study*—Give the eligibility criteria, and the sources and methods of selection of participants | 4 |  |
|  |  | (*b*) *Cohort study*—For matched studies, give matching criteria and number of exposed and unexposed  *Case-control study*—For matched studies, give matching criteria and the number of controls per case |  |  |
| Variables | 7 | Clearly define all outcomes, exposures, predictors, potential confounders, and effect modifiers. Give diagnostic criteria, if applicable | 4 |  |
| Data sources/measurement | 8* | For each variable of interest, give sources of data and details of methods of assessment (measurement). Describe comparability of assessment methods if there is more than one group | 4 |  |
| Bias | 9 | Describe any efforts to address potential sources of bias | Appendix 4 |  |
| Study size | 10 | Explain how the study size was arrived at | 4 |  |

| Quantitative variables | 11 | Explain how quantitative variables were handled in the analyses. If applicable, describe which groupings were chosen and why | 4 |  |
| --- | --- | --- | --- | --- |
| Statistical methods | 12 | (*a*) Describe all statistical methods, including those used to control for confounding | 5 |  |
|  |  | (*b*) Describe any methods used to examine subgroups and interactions | 5 |  |
|  |  | (*c*) Explain how missing data were addressed | 6 |  |
|  |  | (*d*) *Cohort study*—If applicable, explain how loss to follow-up was addressed  *Case-control study*—If applicable, explain how matching of cases and controls was addressed  *Cross-sectional study*—If applicable, describe analytical methods taking account of sampling strategy |  |  |
|  |  | (*e*) Describe any sensitivity analyses | 5 |  |
| **Results** | | | | |
| Participants | 13* | (a) Report numbers of individuals at each stage of study—eg numbers potentially eligible, examined for eligibility, confirmed eligible, included in the study, completing follow-up, and analysed | NA |  |
|  |  | (b) Give reasons for non-participation at each stage | NA |  |
|  |  | (c) Consider use of a flow diagram | NA |  |
| Descriptive  data | 14* | (a) Give characteristics of study participants (eg demographic, clinical, social) and information on exposures and potential confounders | Table 1 |  |
|  |  | (b) Indicate number of participants with missing data for each variable of interest | 6 - 7 |  |
|  |  | (c) *Cohort study*—Summarise follow-up time (eg, average and total amount) | NA |  |
| Outcome data | 15* | *Cohort study*—Report numbers of outcome events or summary measures over time | *6-7* |  |
|  |  | *Case-control study—*Report numbers in each exposure category, or summary measures of exposure |  |  |
|  |  | *Cross-sectional study—*Report numbers of outcome events or summary measures |  |  |
| Main results | 16 | (*a*) Give unadjusted estimates and, if applicable, confounder-adjusted estimates and their precision (eg, 95% confidence interval). Make clear which confounders were adjusted for and why they were included | 6-11 |  |
|  |  | (*b*) Report category boundaries when continuous variables were categorized | 6 |  |
|  |  | (*c*) If relevant, consider translating estimates of relative risk into absolute risk for a meaningful time period | NA |  |

| Other analyses | 17 | Report other analyses done—eg analyses of subgroups and interactions, and sensitivity analyses | 6-11 |  |
| --- | --- | --- | --- | --- |
| **Discussion** | | | | |
| Key results | 18 | Summarise key results with reference to study objectives | 9-10 |  |
| Limitations | 19 | Discuss limitations of the study, taking into account sources of potential bias or imprecision. Discuss both direction and magnitude of any potential bias | 10-11 |  |
| Interpretation | 20 | Give a cautious overall interpretation of results considering objectives, limitations, multiplicity of analyses, results from similar studies, and other relevant evidence | 10-11 |  |
| Generalisability | 21 | Discuss the generalisability (external validity) of the study results | 10-11 |  |
| **Other information** | |  | | |
| Funding | 22 | Give the source of funding and the role of the funders for the present study and, if applicable, for the original study on which the present article is based | NA |  |

**Note:** An Explanation and Elaboration article discusses each checklist item and gives methodological background and published examples of transparent reporting. The STROBE checklist is best used in conjunction with this article (freely available on the Web sites of PLoS Medicine at <http://www.plosmedicine.org/>, Annals of Internal Medicine at <http://www.annals.org/>, and Epidemiology at <http://www.epidem.com/>). Information on the STROBE Initiative is available at [www.strobe-statement.org](https://www.strobe-statement.org).

Appendix 5 – Supplementary Tables and Figures

Contents:

- Supplementary Table S1. Univariable and multivariable predictors of the three key outcomes
- Supplementary Table S2. Firth penalised-likelihood sensitivity analysis for the adverse-event model
- Supplementary Table S3. Extent and mechanism of missing data
- Supplementary Table S4. Sensitivity of satisfaction estimates to alternative missing-data assumptions
- Supplementary Table S5. Procedural success and adverse events by pre-specified procedure group
- Supplementary Figure S1. Adjusted predictors of the three key outcomes
- Supplementary Figure S2. Completeness of outcome documentation by sedation success
- Supplementary Figure S3. Distribution of the deepest UMSS score

## Supplementary Table S1. Univariable and multivariable predictors of the three key outcomes: sedation success, adverse events, and ED length of stay (n=114)

| **Predictor** | **Unadjusted estimate (95% CI)** | **p** | **Adjusted estimate (95% CI)** | **p** |
| --- | --- | --- | --- | --- |
| **Panel A. Sedation success – odds ratio (OR); an OR <1 indicates lower odds of success** | | | | |
| Age, per year | 0.86 (0.74–0.99) | 0.037 | 0.86 (0.74–1.00) | 0.048 |
| Weight, per kg | 0.95 (0.91–0.99) | 0.020 | – |  |
| Dose, per mcg/kg | 0.85 (0.45–1.59) | 0.611 | 0.94 (0.48–1.81) | 0.842 |
| Male sex | 0.96 (0.39–2.35) | 0.933 | 0.88 (0.34–2.25) | 0.786 |
| First Nations status | 1.24 (0.47–3.31) | 0.663 | – |  |
| Concurrent fentanyl | 0.72 (0.18–2.96) | 0.653 | – |  |
| Any medical history | 0.48 (0.18–1.26) | 0.137 | – |  |
| After-hours presentation | 1.14 (0.44–2.93) | 0.792 | – |  |
| Weekend presentation | 0.86 (0.30–2.45) | 0.777 | – |  |
| Painful procedure vs imaging | 0.36 (0.04–3.11) | 0.352 | 0.41 (0.05–3.69) | 0.429 |
| PIVC/other vs imaging | 0.38 (0.04–3.37) | 0.388 | 0.52 (0.06–4.76) | 0.560 |
| **Panel B. Any adverse event – odds ratio (OR); an OR >1 indicates higher odds of an adverse event** | | | | |
| Age, per year | 1.08 (0.91–1.28) | 0.404 | 1.07 (0.90–1.27) | 0.453 |
| Weight, per kg | 1.01 (0.95–1.06) | 0.839 | – |  |
| Dose, per mcg/kg | 1.20 (0.55–2.60) | 0.648 | 1.09 (0.49–2.41) | 0.840 |
| Male sex | 0.67 (0.23–2.00) | 0.474 | 0.71 (0.23–2.16) | 0.541 |
| First Nations status | 0.50 (0.13–1.90) | 0.308 | – |  |
| Concurrent fentanyl | 0.64 (0.08–5.36) | 0.677 | – |  |
| Any medical history | 0.43 (0.09–2.04) | 0.290 | – |  |
| After-hours presentation | 0.96 (0.30–3.02) | 0.939 | – |  |
| Weekend presentation | 3.94 (1.26–12.26) | 0.018 | – |  |
| Painful procedure vs imaging | 0.49 (0.08–2.86) | 0.428 | – |  |
| PIVC/other vs imaging | 0.67 (0.12–3.81) | 0.649 | – |  |
| **Panel C. ED length of stay – % change in geometric mean EDLOS; a positive value indicates a longer stay** | | | | |
| Age, per year | 4.0 (-1.0–9.3) | 0.120 | 2.7 (-2.1–7.8) | 0.273 |
| Weight, per kg | 1.4 (-0.1–2.9) | 0.075 | – |  |
| Dose, per mcg/kg | 14.4 (-6.6–40.2) | 0.192 | 11.7 (-7.8–35.2) | 0.255 |
| Male sex | 3.5 (-22.9–38.9) | 0.819 | – |  |
| First Nations status | -0.6 (-27.5–36.2) | 0.968 | – |  |
| Concurrent fentanyl | -6.9 (-43.3–52.9) | 0.777 | – |  |
| Any medical history | 38.0 (-1.2–92.9) | 0.059 | – |  |
| After-hours presentation | 83.4 (37.6–144.4) | <0.001 | 86.2 (40.2–147.2) | <0.001 |
| Weekend presentation | -11.1 (-37.6–26.6) | 0.511 | – |  |
| Sedation success | -14.2 (-39.7–22.2) | 0.393 | -14.7 (-38.8–18.9) | 0.345 |
| Any adverse event | 18.9 (-22.8–83.2) | 0.429 | 13.7 (-23.7–69.6) | 0.524 |
| Painful procedure vs imaging | -29.0 (-58.3–20.7) | 0.203 | -39.8 (-63.4–-0.9) | 0.046 |
| PIVC/other vs imaging | -7.2 (-45.7–58.7) | 0.784 | -20.9 (-52.3–31.2) | 0.360 |

*CI, confidence interval; ED, emergency department; EDLOS, emergency department length of stay; OR, odds ratio; PIVC, peripheral intravenous cannulation. Panels A and B report odds ratios from logistic regression, with Wald confidence intervals and p-values. Panel C reports the percentage change in the geometric mean EDLOS, obtained by exponentiating coefficients from linear regression on the natural logarithm of EDLOS; confidence intervals and p-values in Panel C are based on the t distribution with 106 residual degrees of freedom. Multivariable models are those pre-specified in the statistical analysis plan and are unchanged from the original submission: Panel A adjusted for age, dose, sex and procedure group; Panel B adjusted for age, dose and sex; Panel C adjusted for age, dose, sedation success, adverse event, procedure group and after-hours presentation. An en dash indicates the variable was not included in the corresponding multivariable model. The reference category for procedure group is medical imaging. No adjustment was made for multiple comparisons; univariable results are exploratory.*

*Model performance: Panel A, area under the receiver operating characteristic curve 0.65, Hosmer–Lemeshow p=0.465; Panel B, likelihood-ratio test against the null model p=0.764, with 15 events for 3 degrees of freedom (5.0 events per variable) and results materially unchanged under Firth penalised likelihood (Supplementary Table S2); Panel C, R² 0.215, adjusted R² 0.163, maximum variance inflation factor 3.52. The single nominally significant univariable association in Panel B (weekend presentation) did not survive Holm correction across the eleven univariable adverse-event tests (adjusted p=0.198) and is reported as a chance finding.*

## Supplementary Table S2. Firth penalised-likelihood sensitivity analysis for the adverse-event model

| **Predictor** | **Maximum-likelihood OR (95% CI)** | **p** | **Firth penalised OR (95% CI)** | **p** |
| --- | --- | --- | --- | --- |
| Age, per year | 1.08 (0.91–1.28) | 0.404 | 1.08 (0.92–1.28) | 0.357 |
| Weight, per kg | 1.01 (0.95–1.06) | 0.839 | 1.01 (0.96–1.06) | 0.702 |
| Dose, per mcg/kg | 1.20 (0.55–2.60) | 0.648 | 1.17 (0.55–2.49) | 0.681 |
| Male sex | 0.67 (0.23–2.00) | 0.474 | 0.68 (0.24–1.96) | 0.474 |
| First Nations status | 0.50 (0.13–1.90) | 0.308 | 0.56 (0.16–1.95) | 0.359 |
| Concurrent fentanyl | 0.64 (0.08–5.36) | 0.677 | 0.88 (0.15–5.33) | 0.891 |
| Any medical history | 0.43 (0.09–2.04) | 0.290 | 0.51 (0.12–2.13) | 0.358 |
| After-hours presentation | 0.96 (0.30–3.02) | 0.939 | 0.99 (0.33–3.02) | 0.992 |
| Weekend presentation | 3.94 (1.26–12.26) | 0.018 | 3.89 (1.29–11.72) | 0.016 |
| Painful procedure vs imaging | 0.49 (0.08–2.86) | 0.428 | 0.45 (0.09–2.28) | 0.332 |
| PIVC/other vs imaging | 0.67 (0.12–3.81) | 0.649 | 0.60 (0.12–3.00) | 0.534 |

*CI, confidence interval; OR, odds ratio; PIVC, peripheral intravenous cannulation. With only 15 adverse events, maximum-likelihood logistic regression estimates are subject to small-sample bias. Firth penalised likelihood removes first-order bias and yields finite estimates under sparse data. Estimates and inferences were materially unchanged, supporting the conclusion that no measured patient or dose characteristic predicted adverse events in this cohort.*

## Supplementary Table S3. Extent and mechanism of missing data (n=114)

| **Variable** | **Missing, n (%)** | **Documented when sedation successful (n=89)** | **Documented when sedation unsuccessful (n=25)** | **p** | **Adjusted OR for non-documentation, per successful sedation (95% CI)** |
| --- | --- | --- | --- | --- | --- |
| Depth of sedation (UMSS) | 24 (21.1) | 73/89 (82.0) | 17/25 (68.0) | 0.165 | 0.54 (0.19–1.52) |
| Clinician satisfaction | 69 (60.5) | 41/89 (46.1) | 4/25 (16.0) | 0.010 | 0.21 (0.06–0.69) |
| Parent/carer satisfaction | 73 (64.0) | 37/89 (41.6) | 4/25 (16.0) | 0.020 | 0.24 (0.07–0.79) |
| Sedation success, adverse events, ED length of stay, age, weight, dose | 0 (0.0) | 89/89 (100.0) | 25/25 (100.0) | – | – |

*CI, confidence interval; ED, emergency department; OR, odds ratio; UMSS, University of Michigan Sedation Scale. p-values are from the Fisher exact test comparing documentation completeness between successful and unsuccessful sedations. The final column gives the odds of an outcome NOT being documented per successful sedation, adjusted for age, dose, sex, after-hours presentation and adverse event. Satisfaction scores were substantially more likely to be documented when sedation was successful, indicating that these data are not missing completely at random; depth of sedation showed no such association. All variables entering the analyses of the three key outcomes were complete.*

## Supplementary Table S4. Sensitivity of satisfaction estimates to alternative missing-data assumptions

| **Assumption** | **Analysis set** | **Mean score (1–5)** | **Satisfied (score ≥4), %** |
| --- | --- | --- | --- |
| Clinician – available case (as reported) | n=45 | 4.27 (SD 1.03) | 80.0 (66.2–89.1) |
| Clinician – missing at random given sedation success (success-standardised) | n=114 | 4.12 | 75.7 |
| Clinician – extreme-case bounds (all undocumented satisfied / dissatisfied) | n=114 | – | 31.6 to 92.1 |
| Parent/carer – available case (as reported) | n=41 | 4.46 (SD 0.84) | 87.8 (74.5–94.7) |
| Parent/carer – missing at random given sedation success (success-standardised) | n=114 | 4.30 | 82.7 |
| Parent/carer – extreme-case bounds (all undocumented satisfied / dissatisfied) | n=114 | – | 31.6 to 95.6 |
| Depth of sedation (UMSS) by sedation success, available case | n=73 successful, n=17 unsuccessful | 2.32 (SD 0.76) vs 1.29 (SD 0.77) | p<0.001 |

*SD, standard deviation; UMSS, University of Michigan Sedation Scale (0 awake and alert to 4 unarousable; higher scores indicate deeper sedation). The available-case analysis is the estimate reported in the manuscript. The success-standardised estimate assumes data are missing at random conditional on sedation success and re-weights observed means and proportions to the full cohort. Extreme-case bounds assume every undocumented response was, respectively, satisfied or not satisfied, and represent the widest range consistent with the observed data. Depth of sedation was deeper in successful sedations, supporting the construct validity of the procedural-success definition. p-value from the Wilcoxon rank-sum test.*

## Supplementary Table S5. Procedural success and adverse events by pre-specified procedure group (n=114)

| **Procedure group** | **n** | **Procedural success, n (%)** | **95% CI** | **Adverse event, n (%)** | **95% CI** |
| --- | --- | --- | --- | --- | --- |
| Medical imaging (non-painful) | 10 | 9/10 (90.0) | 59.6–98.2 | 2/10 (20.0) | 5.7–51.0 |
| Painful procedure | 55 | 42/55 (76.4) | 63.7–85.6 | 6/55 (10.9) | 5.1–21.8 |
| PIVC / other | 49 | 38/49 (77.6) | 64.1–87.0 | 7/49 (14.3) | 7.1–26.7 |

*CI, confidence interval; PIVC, peripheral intravenous cannulation. This is the three-level procedure grouping pre-specified in the statistical analysis plan and used in all regression models, with medical imaging as the reference category. The exact test across the three groups for procedural success gave p=0.795. The seven-category breakdown requested at review is presented in Table 3 of the main manuscript.*

## Supplementary Figure S1. Adjusted predictors of the three key outcomes (n=114)

*Forest plot of the multivariable (adjusted) estimates reported in Supplementary Table S1. Axis convention: Panels A and B report a ratio measure (the odds ratio), whose null value is 1, and are plotted on a symmetric logarithmic axis centred on 1 so that equal and opposite effects (for example OR 0.25 and OR 4.0) receive equal visual weight; a linear axis anchored at zero would compress the entire protective range into a narrow band and visually exaggerate harmful effects. Panel C reports a difference measure (percentage change in the geometric mean ED length of stay), whose null value is 0, and is plotted on a linear axis centred on zero. Count and percentage axes elsewhere in this supplement are anchored at zero.*

## Supplementary Figure S2. Completeness of outcome documentation by sedation success

*Proportion of sedations for which each outcome was documented, by whether the sedation was successful. Fisher exact test: depth of sedation p=0.165; clinician satisfaction p=0.010; parent/carer satisfaction p=0.020. The proportion axis is anchored at zero. Satisfaction was recorded roughly three times as often when sedation had succeeded, indicating that these data are not missing completely at random.*

## Supplementary Figure S3. Distribution of the deepest UMSS score

*University of Michigan Sedation Scale (UMSS), recorded as the deepest level reached during the sedation episode: 0 awake and alert, 1 minimally sedated, 2 moderately sedated, 3 deeply sedated, 4 unarousable. A score was documented for 90 of 114 sedations (78.9%); mean 2.1 (SD 0.9). The count axis is anchored at zero and the scale is displayed across its full 0–4 range so that sparsely populated levels remain visible.*
